# Mode of inhibitory binding of epigallocatechin gallate to the ubiquitin-activating enzyme Uba1 *via* accelerated molecular dynamics†

**DOI:** 10.1039/d0ra09847g

**Published:** 2021-02-22

**Authors:** Paras Gaur, Gabriel Fenteany, Chetna Tyagi

**Affiliations:** Institute of Genetics, Biological Research Centre Temesvári krt. 62 6726 Szeged Hungary; Doctoral School of Biology, Faculty of Sciences and Informatics, University of Szeged Közép fasor 52 Szeged 6726 Hungary chetna.tyagi@bio.u-szeged.hu; Department of Microbiology, Faculty of Science and Informatics, University of Szeged Közép fasor 52 6726 Szeged Hungary

## Abstract

The green tea polyphenol (−)-epigallocatechin-3-gallate (EGCG) and some of its analogs potently inhibit the ubiquitin-activating enzyme Uba1. In an effort to understand the possible molecular basis of inhibitory activity of EGCG, we conducted a molecular docking and molecular dynamics simulation study. We found that EGCG and its two selected analogs, (−)-epicatechin-3-gallate (ECG) and (−)-epigallocatechin (EGC), bind favorably at two likely hot spots for small-molecule ligand binding on human Uba1. The compounds bind with energetics that mirror their experimental potency for inhibition of Uba1∼ubiquitin thioester formation. The binding of EGCG, ECG, and EGC at one of the hot spots, in particular, recapitulates the rank order of potency determined experimentally and suggests a possible mechanism for inhibition. A hinge-like conformational change of the second catalytic cysteine domain and the opposing ubiquitin-fold domain observed during accelerated molecular dynamics simulations of the EGCG-bound Uba1 complex that results in disruption of the ubiquitin-binding interfaces could explain the compounds' inhibitory activity. These results shed light on the possible molecular mechanism of EGCG and related catechins in the inhibition of Uba1.

## Abstract 2

The hinge-like movement of the SCCH domain upon ligand binding closes the ubiquitin binding site and disrupts the interfaces crucial for thioester bond formation.

## Graphical abstract

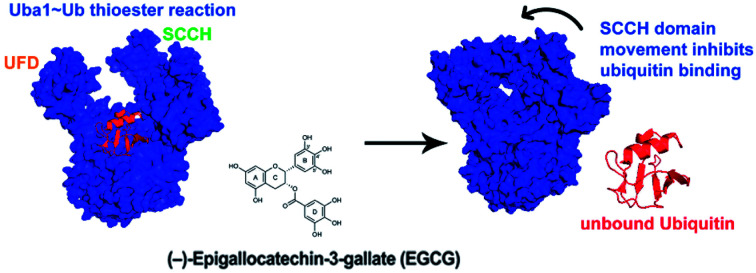

## Introduction

In the process of screening for small-molecule modulators of a completely reconstituted ubiquitination cascade that helps trigger the DNA damage response, we discovered that the plant polyphenol (−)-epigallocatechin-3-gallate (EGCG) and certain of its analogs are potent inhibitors of the ubiquitin-activating enzyme 1 (Uba1 or UBE1) *in vitro*, as well as in the cell.^1,2^ Ubiquitination controls a myriad of other pathways, most notably proteasomal degradation, by serving as a tag or composite docking site for recruiting other proteins to form complexes that regulate an enormous range of other processes and it is a major focal point in drug discovery and development in addition to research probe discovery (reviewed in ref. 3–9).

Uba1 is an E1 ubiquitin-activating enzyme that lies at the head of the bulk of ubiquitination cascades (another E1 enzyme, Uba6, also has similar activity but it is only responsible for a tiny minority of ubiquitination events); Uba1 may also be a viable therapeutic target for the treatment of cancers, neurodegenerative disorders, and other disease states (reviewed in ref. 10–14). Uba1 activates ubiquitin by first catalyzing reaction of the C-terminal glycine's carboxylate with ATP, then nucleophilic attack on the resultant ubiquitin adenylate by the active-site cysteine of Uba1 to yield a high-energy Uba1∼ubiquitin thioester conjugate. The ubiquitin moiety then undergoes transthioesterification to an E2 ubiquitin-conjugating enzyme, of which approximately 40 are encoded in the human genome. Finally, in conjunction with an E3 ubiquitin ligase, for which more than 600 genes exist in the human genome, the ubiquitin is transferred to the ε-amino group on the side chain of specific lysine residues on the ultimate substrates, conjugated by an isopeptide bond. This linkage is generally stable in the absence of deubiquitinating enzymes (reviewed in ref. 6 and 15).

Therapeutic intervention in the ubiquitin–proteasome pathway has become a major focus of drug discovery and development efforts, particularly, in light of the efficacy of proteasome inhibitors such as bortezomib (Velcade) in the treatment of multiple myeloma. However, inhibitors of Uba1 itself in the upstream ubiquitination component of this pathway and the enormous number of other processes controlled by ubiquitination are currently limited in number. The pyrazolidines PYR-41 ^13^ and PYZD-4409 ^14^ have been shown to bind and irreversibly inhibit Uba1. The adenosine sulfamate TAK-243 (formerly known as MLN7243) has been found to potently inhibit Uba1 and is of significant clinical interest.^16^ TAK-243 is a potent mechanism-based inhibitor of the formation of the Uba1∼ubiquitin thioester through irreversible reaction with ubiquitin's C-terminal carboxylate. TAK-243 and other reactive adenosine derivatives have also been found to similarly inhibit indirectly other ubiquitin-like protein-activating enzymes to different extents.^16–19^ PYR-41 is considerably less potent as a cytostatic/cytotoxic agent than TAK-243 in cells, but PYR-41 instead directly inactivates Uba1 itself rather than directly targeting ubiquitin.^13^ TAK-243 and PYR-41 also differ in some of their biological effects.^20^ In addition, a few other synthetic molecules,^21,22^ natural products,^23–25^ and modified ubiquitin derivatives^26^ have been reported to inhibit Uba1. We discovered through *in vitro* and cellular structure–activity relationship (SAR) profiling that EGCG and certain analogs represent new potent Uba1 inhibitors by directly binding Uba1 and reversibly blocking the formation of the Uba1∼ubiquitin thioester, thus, inhibiting ubiquitination *in vitro* and in the cell.^1,2^ In addition to EGCG, we chose two analogs of the experimentally examined for the present computational study, (−)-epicatechin-3-gallate (ECG), and (−)-epigallocatechin (EGC), the last lacking the gallate ester moiety of the first two. These compounds inhibit formation of this adduct in the rank order by half-maximal inhibitory concentration (IC_50_) values of EGCG > ECG > EGC, with the potency of EGCG and ECG being close to each other.^1,2^

The E1 protein Uba1 is a multidomain enzyme (reviewed in ref. 11 and ^12^). The human Uba1 (hUba1) structure shares many similarities with the *Saccharomyces cerevisiae* and *Schizosaccharomyces pombe* orthologs.^27–29^ Uba1 has an inactive adenylation domain (IAD) and active adenylation domain (AAD) which associate to form a pseudodimeric adenylation domain that serves as a rigid body of the overall structure. The first catalytic cysteine (FCCH) domain connected to the AAD through two loops called β7 and β14 loops. The second catalytic cysteine domain (SCCH) is connected to the AAD through two loops known as crossover and reentry loops, respectively. The ubiquitin-fold domain (UFD) is connected to the AAD by a crossover loop, all of which play important roles in Uba1 function by providing interfaces for ubiquitin binding and stability. Taken together, the domain organization of Uba1 gives the protein a Y-shaped structure with the pseudodimeric adenylation domain forming the base of the enzyme. The SCCH and UFD are situated across from each other at the “top” of the enzyme, with a large gap between them that accommodates the E2 ubiquitin-conjugating enzyme during the E1–E2 ubiquitin thioester transfer step in ubiquitination cascades.

**Fig. 1:**
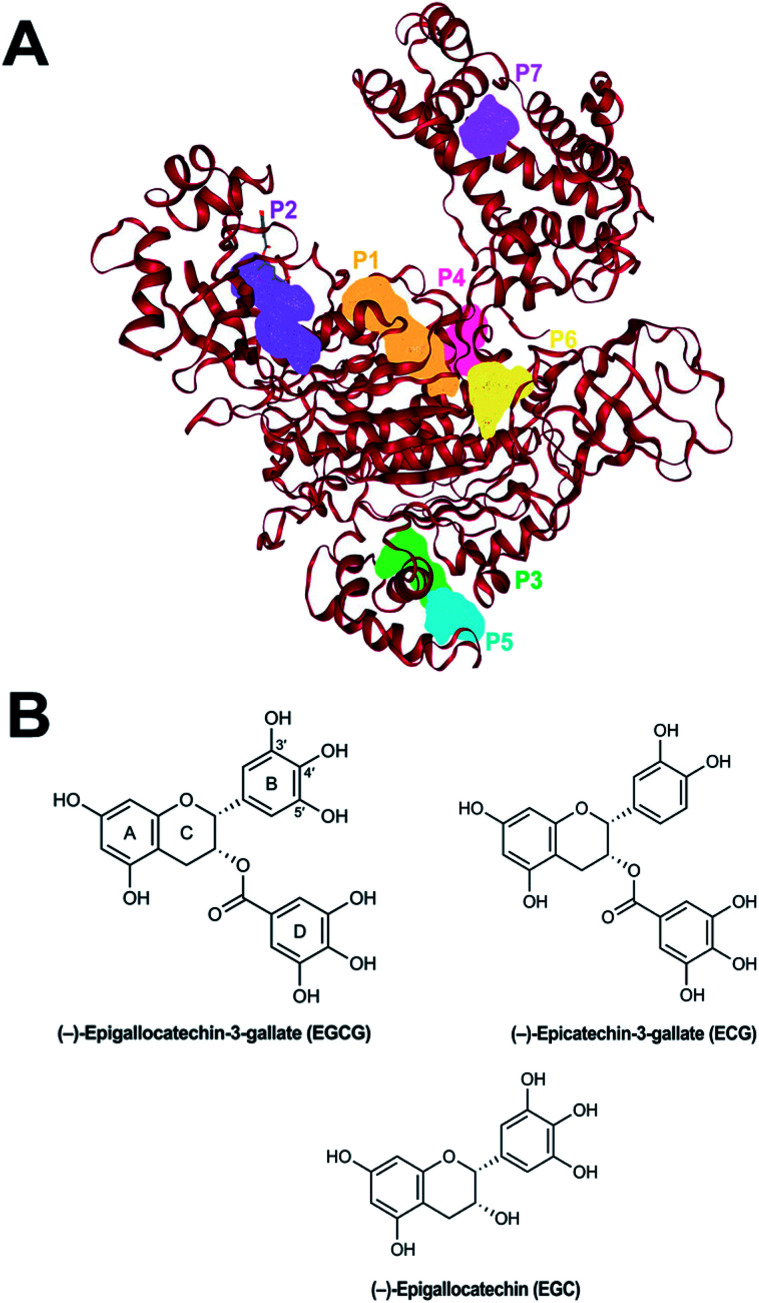
(A) The top seven scoring putative binding sites of human Uba1 protein calculated using DoGSiteScorer. (B) Chemical structure of EGCG.

FTMap analysis of the Uba1 structure suggests four possible hot spot (HS) pockets that can potentially be used for the design of small-molecule inhibitors of Uba1.^28^ The highest scoring HS is HS1, which corresponds to the ATP-binding site. HS2 is located between the UFD and AAD which is in the proximity of where E2 proteins bind during the transthioesterification reaction. HS3 is formed by residues from α-helices H19, H20, H22, H23, and H25 on the SCCH domain, while HS4, is defined by residues from the β5 strand, H7, the β4–H5 loop, and the H7–H8 loop at the bottom of the IAD (Fig. 1). In the present study, we deployed a computational docking approach to further understand the possible mechanism of action of the catechins EGCG, ECG, and EGC at these putative HS regions. We found that docking to HS2 recapitulates the experimental SAR data, with a rank order of binding energies of EGCG > ECG > EGC, while binding to HS3 nearly does so, with an order of ECG > EGCG > EGC.

## Methods

### Binding site detection and analysis

Based on a previous study on the structure of human Uba1 along with four possible ligand-binding hot spots as calculated by the FTMap server,^28^ we chose to check the binding affinity of three selected catechins—EGCG, ECG, and EGC—on all four possible binding sites of human Uba1 (PDB code: 6DC6). DoGSiteScorer server is a quick automated pocket detection method, which is a grid-based method that uses a Difference of Gaussian filter.^30,31^ Algorithmic pocket detection in the hUba1 protein for possible binding sites has led to the detection of more than the four previously reported sites by Lv *et al.*^28^ due to parsing of every cavity that could harbor an approaching ligand. The three spots in between them are occupied by sites close to HS1 and HS4. The HS1 site is large enough to accommodate the C-terminus of a ubiquitin molecule and presents interfaces to stabilize this complex which may explain more than one plausible binding site around HS1.

### Extra-precision (XP) docking of the three catechins to hUba1 with Schrodinger's glide

The site-specific docking of catechins to Uba1 was carried out by forming a cubic grid (20 Å^3^) around the selected residues of each hotspot with the “Receptor Grid Generation” platform of Schrödinger's Glide module. The three ligands were prepared for docking by 2D to 3D molecular conversion with the LigPrep module using the default OPLS3e force field. All docking calculations were carried out with the XP protocol available in the Glide module.

### Accelerated molecular dynamics simulations of the Uba1–EGCG complex

To understand the dynamics of ligand binding with Uba1, accelerated molecular dynamics simulation (aMD) was carried out for a total time of 300 ns. The ligand EGCG was parameterized using *antechamber* and the whole complex PDB file outputs from Schrödinger was stripped of all H atoms. Both systems were solvated with TIP3P water at a cutoff of 12.0, which added 46 768 water residues with cubic box of size of 120.65 × 122.75 × 124.00 Å and a volume of 1 836 550.27 Å^3^ in both complexes. The initial preparation of protein–ligand complexes for Amber simulation caused a renumbering of residues to 1–992 instead of 1–1057 as in the original structure downloaded from the PDB.

The solvated Uba1–EGCG complex system was prepared for aMD in six consecutive steps by a previously published operation.^32^ Berendsen barostat and Langevin thermostat were used for pressure and temperature scaling, respectively. SHAKE bond length constraints were applied to all bonds involving hydrogen. A short molecular dynamics run for 450 ps was also carried out for each aMD run to calculate the torsional and total energy boost parameters.

Following our previously published procedure,^32^ for each aMD simulation, particle mesh Ewald summation (PME) was used to calculate the electrostatic interactions. Long-range interactions were calculated with a cutoff of 10.0. The simulations were carried out at 300 K temperature and 2 fs time step. The National Information Infrastructure Development clusters of the University of Debrecen, Hungary were sourced for running simulations on GPUs with the *pmemd.cuda* implementation of Amber14. The aMD simulations required extra parameters *E*_dihed_, *α*_dihed_, *E*_total_, and *α*_total_ which can be calculated using eqn (1):

1 *E*_dihed_ = *V*_avg_dihed_ + *a*_1_ × *N*_res_, *α*_dihed_ = *a*_2_ × *N*_res_/5

*E*_total_ = *V*_avg_total_ + *b*_1_ × *N*_atoms_, *α*_total_ = *b*_2_ × *N*_atoms_where *N*_res_ is the number of peptide residues (992 residues) and *N*_atoms_ is the total number of atoms in the system, which is 140 653 in the Uba1–EGCG system. *V*_avg_dihed_ and *V*_avg_total_ are the average dihedral and total potential energies obtained from the classical MD run. The values of coefficients *a*_1_ and *a*_2_ were chosen to be 4 kcal mol^−1^ and *b*_1_ and *b*_2_ were chosen to be 0.16 kcal mol^−1^ based on a previous study.^33^ The energy and boost information was saved at each 1000 time-step.

The dihedral based PCA was carried out using the *cpptraj* module.^34^ For dihedral PCA, the *Φ* and *Ψ* torsion angles are calculated for all residues and the covariance matrix is calculated. The eigenvectors were calculated based on the covariance matrix. The first two principal components are reweighted by the Maclaurin series expansion method. *Grcarma*^35,36^ was used to generate the highest populated clusters using the top three principal components (PC) and write their representative structures in pdb format files.

### MM/PBSA-based estimation of Δ*G*_bind_ energies of EGCG at HS2 and HS3

The MM/PBSA (molecular mechanics energies combined with the Poisson–Boltzmann or generalized Born and surface area continuum solvation) approach was used to estimate the binding free energy, Δ*G*_bind_, using the Amber-compatible Python script *MMPBSA.py*. It is used to calculate the free energy difference between two states, for example, bound or unbound states of a protein or even two different conformations of the same protein as shown in eqn (2):

2 Δ*G*_bind_ = 〈*G*_PL_〉 − 〈*G*_P_〉 − 〈*G*_L_〉

where P stands for protein and L for ligand. The free energy of a state whether P, L or PL is estimated using eqn (3):

3 *G* = *E*_bind_ + *E*_el_ + *E*_vdW_ + *G*_pol_ + *G*_np_ − *TS*

where *E*_bind_, *E*_el_, *E*_vdW_ are energy terms from bonded, electrostatic and van der Waals interactions, respectively. *G*_pol_ and *G*_np_ are the polar and non-polar contributions to solvation free energies. The Poisson–Boltzmann surface area (PBSA) equation was used to calculate *G*_pol_. The last term is the absolute temperature *T* and the entropy *S*.

## Results

### Binding site (hot spots) and their properties

It has been reported that hUba1 has four possible hot spots for small-molecule binding as potential inhibitors by employing the FTMap server.^28^ We confirmed the possible binding sites with another algorithm, DoGSiteScorer. The largest and top-scoring binding site is the same as HS1 lined by 25 donor and 38 acceptor residues with a volume of 575.70 Å^3^, a surface area of 670.29 Å^2^, and a depth of 22.45 Å. The second largest cavity was predicted to be similar to HS2 lined by 12 H-bond donors and 36 acceptors with a volume of 494.82 Å^3^, a surface area of 639.48 Å^2^, and a depth of 11.32 Å. The third-ranking site, however, corresponds to HS4 lined by 10 donor residues and 16 acceptor residues with a much smaller volume of 268.13 Å^3^, a surface area of 618.36 Å^2^, and a depth of 14.19 Å. It is placed higher due to higher druggability score. The site corresponding to HS3 is ranked at 7th position due to a smaller volume of 221.12 Å^3^, a surface area of 221.76 Å^2^, and a depth of 11.81 Å, and lined by only two donor residues and 16 acceptor residues. Positions 4–6 are cavities found close to other hot spots with higher volume and donor residues than HS3, which increases their druggability. In the 20 Å cubic grid used for docking, the neighboring sites 4–6 are automatically included within the top four hotspots. The top seven scoring binding sites are shown in Fig. 1A, while the structures of EGCG with numbered rings and ECG and EGC are provided in Fig. 1B.

### Mode of binding of the catechins to hUba1: comparison of four potential binding sites

**Fig. 2:**
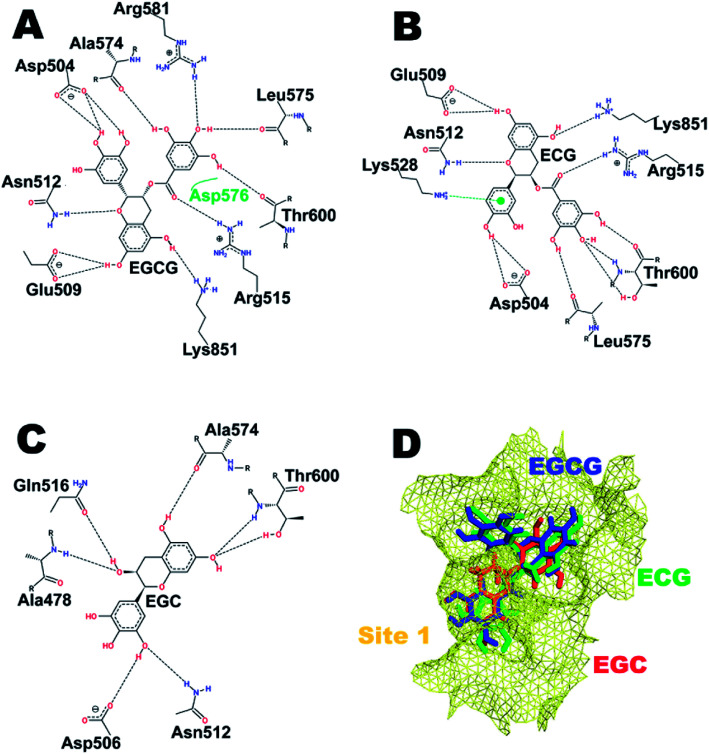
Interaction pattern of catechins at hot spot (HS) 1. (A) EGCG. (B) ECG. (C) EGC. (D) Comparative binding poses of the three catechins at the ATP-binding site.

The three catechins EGCG, ECG, and EGC were consecutively docked at the four potential binding sites or hot spots as mentioned earlier. Their respective binding scores in kcal mol^−1^ are listed in Table 1. Fig. 2 shows the interacting residues of HS1 with all three catechins. Fig. 2D depicts the comparative binding pose at the ATP-binding site of Uba1. It is clear that EGCG and ECG have almost identical binding poses with the ring of the gallate ester (D-ring) embedded deeper in the cavity, forming bonds with Ala574, Leu575, and Thr600, while the connecting O atom of the D-ring forms an H-bond with Arg515 in both the cases. Glu509, Asn512, Asp54, and Lys851 also form H-bonds with EGCG and ECG in a similar manner with the same atoms except for also involving another residue, Lys528, which forms a pi-cation interaction with ECG but not with EGCG, probably due to the absence of one hydroxyl group in the former, providing larger space for the interaction to take place. It has previously been shown that Lys528 and Asp576 are critical for substrate binding,^37^ and interaction of ECG with Lys528 may explain its highest binding score (Table 1). EGC, on the other hand, due to the absence of a gallate ester moiety, enters the cavity lined by Ala574 and Thr600 and forms the least number of H-bonds, which is reflected in its low binding score.

**Table 1:** Binding scores in kcal mol^−1^ 1 for EGCG, ECG, and EGC binding to the four hot spots of Uba1

Binding site	EGCG (kcal mol^−1^)	ECG (kcal mol^−1^)	EGC (kcal mol^−1^)
HS1	−7.49	−9.53	−7.42
HS2	−8.137	−7.94	−7.3
HS3	−9.58	−9.89	−6.46
HS4	−7.37	−7.81	−8.45

**Fig. 3:**
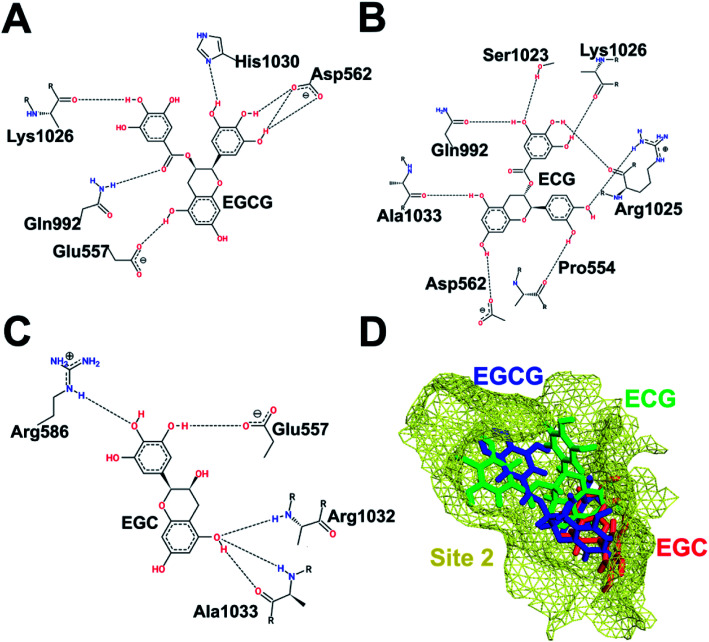
Interaction pattern of catechins at HS2. (A) EGCG. (B) ECG. (C) EGC. (D) Comparative binding poses of the three catechins at HS2.

Similarly, Fig. 3 shows the binding of the three catechins with HS2 (junction between UFD and AAD domain) of Uba1, which represents the surface that interacts with E2s with extended N-termini. Here, the relative binding poses of EGCG and ECG differ to a greater extent than in HS1. The whole catechin moiety of EGCG is embedded inside the cavity lined by Glu557, Asp562, and His1030, while the catechin moiety of ECG is slightly out of the cavity and makes H-bonds with Pro554, Asp562, Arg1025, and Ala1033. Lys1026 interacts with the same aromatic ring in both ligands while Asp562 and Gln992 are common interacting partners but with different H-bonding patterns. EGC is completely embedded inside the cavity and interacts with Glu557, Arg586, Arg1032, and Ala1033. The differences in the binding poses of the three molecules highlight the importance of the D-ring in forming stable interactions. The B-ring of ECG protrudes outwards in contrast to EGCG due to the interaction with Arg1025, which also makes an H-bond with the D-ring of ECG. This double interaction of Arg1025 with two aromatic rings of ECG pushes them closer to each other (Fig. 3D).

**Fig. 4:**
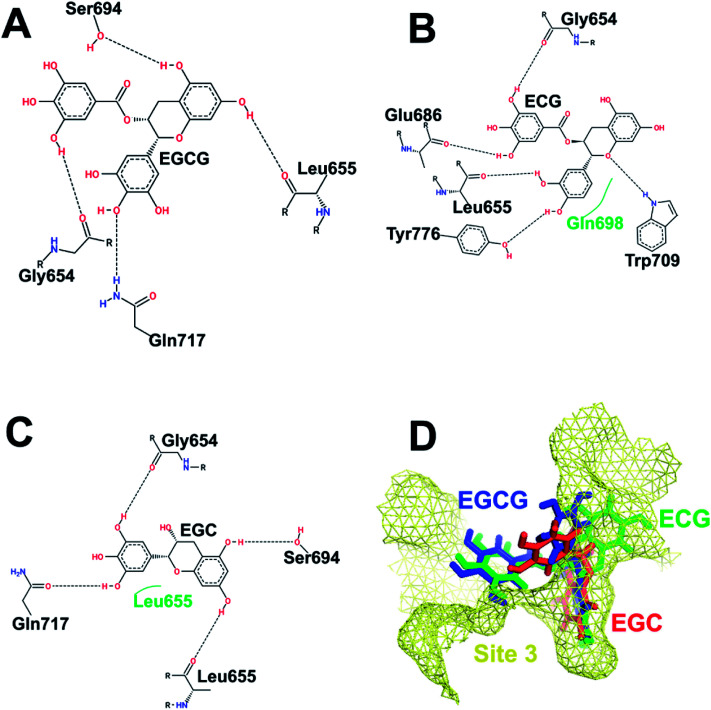
Interaction pattern of catechins at HS3. (A) EGCG. (B) ECG. (C) EGC. (D) Comparative binding poses of the three catechins at HS3.

Fig. 4 depicts the binding of the three catechins with HS3 (helices H19, H20, H22, H23, and H25 on the SCCH domain but far from the catalytic cysteine) of Uba1. The gallate ester moiety (D-ring) of both EGCG and ECG occupies the same space and makes an H-bond with Gly654 which is otherwise, transferred to the B-ring in EGC. Similarly, the A-ring and C-ring of both EGCG and EGC show the same H-bonding pattern with Leu655 and Ser694. Despite similarities in H-bonding patterns, the docking score of EGCG is much higher than EGC which can be explained by the interaction of Gly654 with the D-ring in the former instead of the B-ring as in the latter. Both EGCG and ECG display an interaction with Gly654 in the same manner and their docking scores are similar. This observation also highlights the importance of the D-ring in the interaction.

**Fig. 5:**
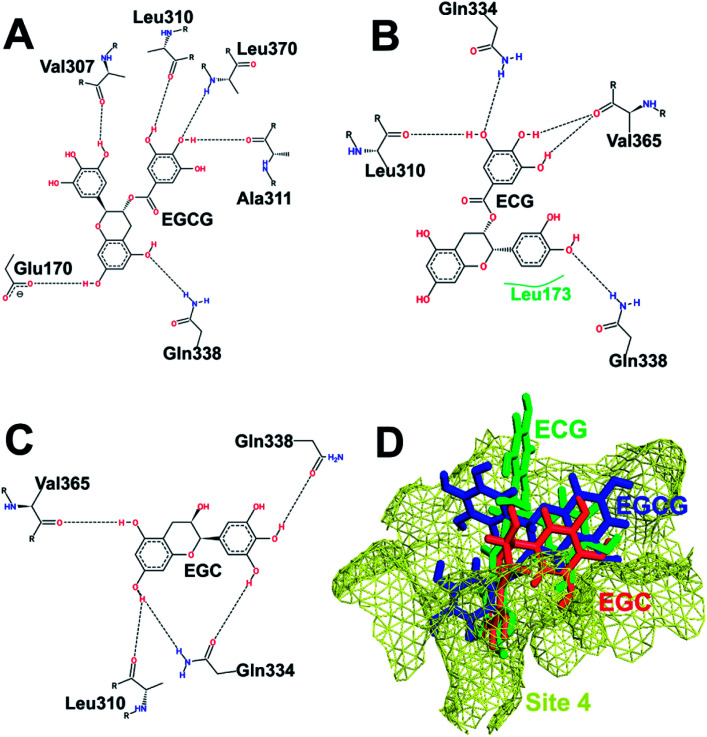
Interaction pattern of catechins at HS4. (A) EGCG. (B) ECG. (C) EGC. (D) Comparative binding poses of the three catechins at HS4.

Fig. 5 shows the binding of the three catechins with HS4 (bottom of the IAD domain) of hUba1. This site is far away from the active site or the E2 binding site but was recently reported to be partially occupied by the Uba1 inhibitor NSC 624206 in an X-ray co-crystal structure of *S. pombe* Uba1 (PDB code: 5UM6).^27^ All three catechins bind with different poses at HS4, although the residues in interaction with ECG and EGC are the same, just with different H-bonding patterns. Here, the presence of the D-ring results in lowering of the binding score, which is in contrast with our previous experimental results and, thus, was not pursued for further analysis.

The binding scores at HS1 show the pattern ECG > EGCG > EGC while the scores at HS4 show the pattern EGC > ECG > EGCG. Binding scores at HS2 seems to follow the experimental pattern of bioactivity with a rank order of EGCG > ECG > EGC,^1,2^ while binding scores at HS3 are the highest amongst all four hot spots in the order ECG > EGCG > EGC. The experimental bioactivities of EGCG and ECG are so similar^1,2^ that we can consider both HS2 and HS3 as plausible.

### Accelerated molecular dynamics to elucidate conformational dynamics of hUba1 upon binding EGCG

**Fig. 6:**
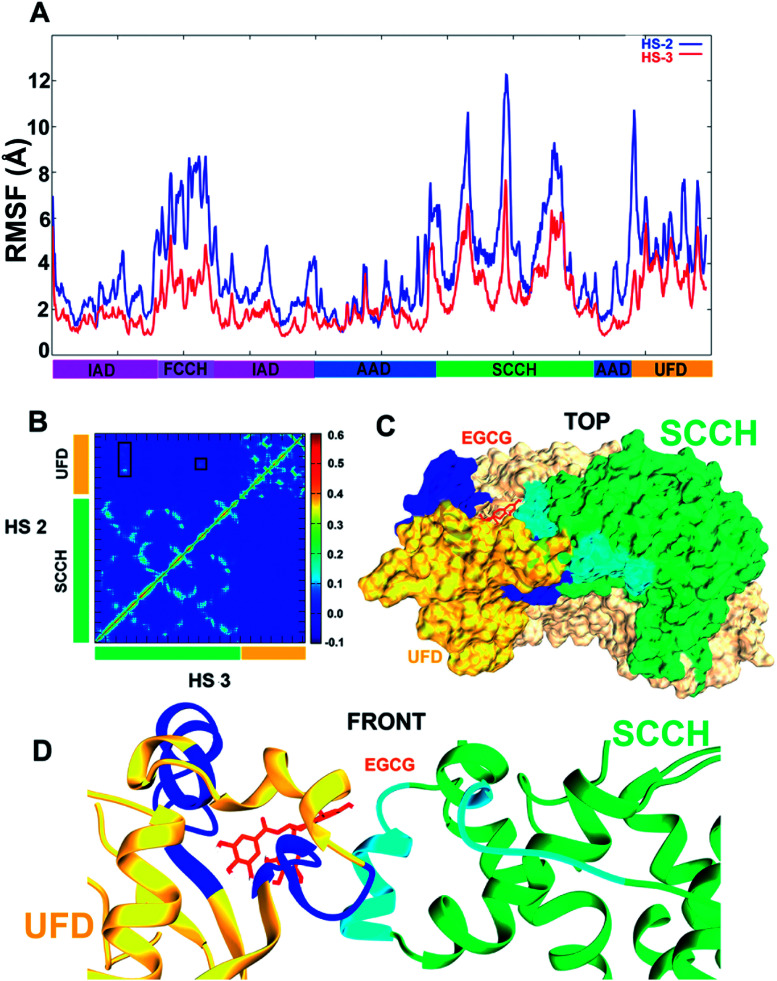
Structural comparison between EGCG binding at HS2 and HS3. (A) The root-mean-square fluctuation (in Å) calculated or each amino acid residue of hUBA1 with EGCG bound at HS2 and at HS3. EGCG binding at HS2 introduces higher degree of atomic fluctuations in the whole protein with FCCH, SCCH and UFD domains with largest deviations. (B) A heat map of the motional correlations between the SCCH and UFD domains; top left depicting when EGCG binds at HS2 and bottom left when it binds at HS 3. A positive correlated movement is observed between Ser991–Met1002 and Ile1018–Leu1036 of UFD domain with Pro670–Leu679 and Val821–Leu826 of the SCCH domain. (C) A top-view surface representation of the post-simulated EGCG-hUBA1 complex that results in closing of the canyon region; the dark blue and light blue are the interacting residues of the UFD and SCCH domains, respectively. (D) A front-view cartoon representation of post-simulated EGCG-hUBA1 complex which shows interacting UFD and SCCH domains.

Owing to results discussed in the previous section, two aMD simulations were set up with EGCG in complex with hUba1 at HS2 and at HS3 to compare dynamic evolution of conformational change that occurs in the protein upon ligand binding. It is clear that EGCG binding at HS2 increases the root-mean-square fluctuation (RMSF) of the whole protein in comparison to EGCG binding at HS3 (Fig. 6A). It simply means that binding of EGCG at HS2 increases the overall fluctuation of the Uba1 protein in comparison to its binding at HS3. In terms of domain-wise fluctuation, the FCCH, SCCH, and UFD domains show the highest value amongst all domains, indicating a conformational perturbation upon EGCG binding. A rotation of SCCH domain observed during simulations to achieve a “closed” conformation can be compared to its similar movement during thioester bond formation with the approaching ubiquitin molecule. The resultant disruption of contacts between the FCCH and SCCH domain may explain the high atomic fluctuation of the FCCH domain.

The average correlations between motions of amino acid residues were calculated between the SCCH and UFD domains (Fig. 6B). Every frame has been considered to calculate a motion vector for every amino acid residue from its previous position to its present position. The value ranges from 1.0 for residues showing correlated motion, to 0.0 for no correlation, and to −1.0 for anticorrelated motions. The top left diagonal of the contour plot shows the correlation of motion between the two domains upon binding of EGCG at HS2 (UFD), while the bottom right diagonal describes that correlation upon EGCG binding at HS3 (SCCH). As apparent from the plot, no motional correlation could be observed for EGCG bound at HS3 complex while a small positive correlation is observed in the motions of residues Ser991–Met1002 and Ile1018–Leu1036 of UFD domain with Pro670–Leu679 and Val821–Leu826 of the SCCH domain marked with rectangles (Fig. 6B). It means that the residues belonging to the SCCH and UFD domains move in correlation to each other during the conformational change that takes place due to EGCG binding at HS2. This interaction is shown with protein surfaces in Fig. 6C, with the dark blue representing interacting residues from the UFD domain and light blue representing interacting residues of the SCCH domain. The position of EGCG bound at HS2 is clear from Fig. 6D, where the SCCH domain moves inward towards UFD domain, thereby arriving at a closed conformation of the Uba1. Such a strongly correlated motion between these domains that lie opposite to each other can be seen only when EGCG binds at HS2 which suggests that its higher bioactivity^1,2^ is related to protein conformational change. Even though its binding score is slightly higher when bound to HS3, no comparable domain movement that would lead to a closed conformation of Uba1 could be observed during the simulation.

**Fig. 7:**
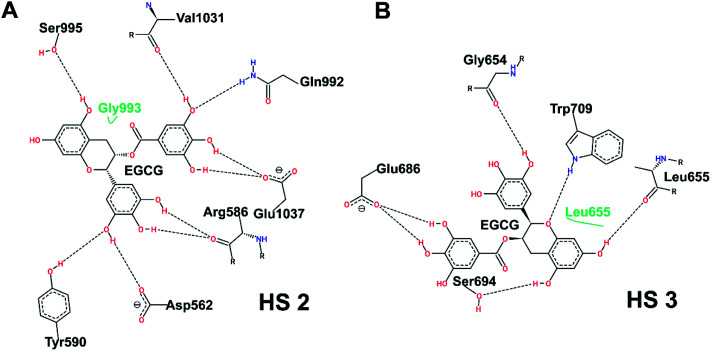
Post-simulation interaction pattern of EGCG at HS2 (A) and at HS3 (B).

Fig. 7 shows the post-simulation binding pattern of EGCG at HS2 and HS3. When compared with the pre-simulation interaction pattern, only Asp562 and Gln992 are the common residues, while Arg586, Tyr590, Ser995, Val1031, and Glu1037 present new H-bonds that demonstrate that EGCG acquires a new binding pose and shifts in the binding cavity during the simulation (Fig. 7A). A superimposed representation of the pre- and post-aMD simulated binding pose of EGCG at the HS2 cavity shows that the D-ring moves deeper inside the cavity during simulation (ESI Fig. 1A†). A comparison of the binding site before and after conformational change shows the shift in the binding residues as EGCG moves deeper into the cavity. The Glu1037 side-chain moves in closer to the D-ring that may explain strong binding and importance of the role of D-ring. Moreover, the His1030–Leu1034 patch also shifts considerably from its position bringing new interactions with EGCG while the shift of Arg586 results in 2 new H-bonds (ESI Fig. 1B†).

On the other hand, a comparison of the interaction pattern at HS3 shows that three out of four H-bonds are preserved with Gly654, Leu655, and Ser694, while two new bonds are formed with Glu686 and Trp709 which shows that the D-ring shifts during the simulation (Fig. 7B). A much larger variation in the root-mean-square deviation (RMSD) of EGCG bound at HS2 could be observed during the course of simulation in comparison to EGCG bound at HS3 which becomes stable after 20 ns (ESI Fig. 1C†). This indicates that a larger conformational change occurs not only in the Uba1 protein upon EGCG binding at HS2 but also in the binding pose of the ligand itself. While EGCG at HS3 seems to be tightly bound indicated by higher docking scores and the complex is quickly stabilized, this does not lead to large conformational shifts.

### Dihedral principal component analysis to retrieve highly occurring hUba1–EGCG complex states

**Fig. 8:**
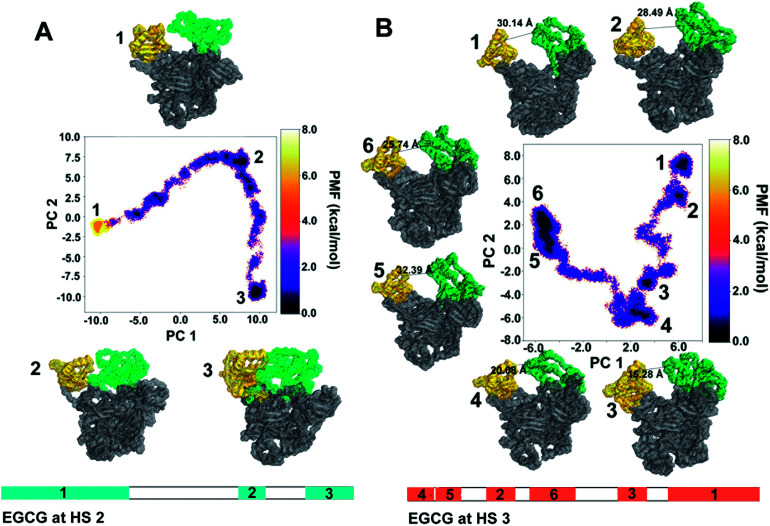
A dihedral angle PCA based free energy landscape of Uba1 when EGCG binds (A) at HS2 (B) at HS3. The corresponding representative conformations have been marked with their respective cluster numbers while the distances in Å between the SCCH and UFD domains have also been marked at (B). The structural clusters obtained during the two simulations have been represented as a function of time.

The first two principal components, PC1 and PC2, were plotted as free energy landscapes after reweighting with the Maclaurin series expansion method, and the various representative states obtained from each energy clusters were obtained. Fig. 8A represents a two-dimensional free-energy landscape of Uba1 when EGCG binds at HS2 between the AAD and UFD domains. This binding renders the usual state of Uba1 energetically unstable as represented by conformation 1. The Uba1 protein undergoes a marked hinge-like movement of the SCCH domain that brings the UFD and SCCH domains closer to each other as discussed before, thereby finally closing the ubiquitin-binding site as represented by conformation 3. This closed Uba1 conformation is the most energetically stable one obtained through the simulation while the open conformation 1 is separated by a ∼5 kcal mol^−1^ energy barrier. A diagrammatic representation of the clusters obtained during aMD simulation for both complexes is also depicted. For EGCG at HS2, cluster 1 occurs for the longest simulation time of 105 ns, preceded by cluster 2 occurring from 200 to 226 ns which denotes the intermediate conformation 2 from Fig. 8A. The last, cluster 3, occurs from 264 ns until the end of simulation at 300 ns which denotes the closed conformation 3 from Fig. 8A.

On the other hand, when EGCG binds at HS3 on Uba1, a similar hinge-like movement of SCCH domain can be observed even though it is not as drastic as observed for HS2 (Fig. 8B). The distance between the UFD and SCCH domains has also been marked for all conformers. The conformational dynamics starts from cluster 1 with 30.14 Å distance between UFD and SCCH which reduces to 15.28 Å until it reaches conformation 3. From this point, the inter-domain distance starts to increase again as seen for conformations 5 and 6. All these conformations lie at the same energy level and are energetically stable. This simple hinge-like motion can be accessed within ∼2 kcal mol^−1^ and is comparable to the distal and proximal conformations defined for Uba1 in a previous study.^38^ Similarly, for EGCG binding at HS3, 6 clusters were obtained from the 300 ns long aMD simulation. Clusters 1 and 2 appear very close to each other with the former obtained until 24 ns and the latter obtained from 26 to 42 ns. Cluster 3 appears from 74 to 94 ns and cluster 4 follows it from 114 to 138 ns. Finally, clusters 5 and 6 were obtained from 182 to 212 ns and from 220 to 300 ns, respectively, and form the largest group.

This analysis shows that EGCG binding at HS3 does not bring a significant conformational change in Uba1 protein as compared to its binding at HS2.

**Fig. 9:**
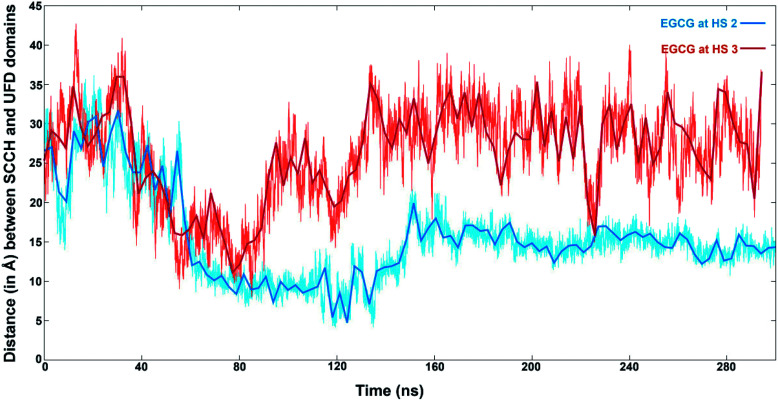
Structural dynamics observed for hUba1 upon EGCG binding (A) Distance (in Å) between the SCCH and UFD domains calculated during the course of simulation with EGCG bound at HS2 (in blue) and bound at HS3 (in dark red).

The distances between the two domains have been calculated by the distance between the Cα atoms of Leu679 from SCCH and Met1007 of the UFD domain (Fig. 9). EGCG bound at HS2 results in a sharp decrease in this distance at the 60 ns time step and remains between 15 to 20 Å for the rest of the simulation. On the other hand, when EGCG binds to HS3, a sharp decrease in this distance is observed between 40 to 80 ns time frames but increases again and remains much higher than observed for EGCG bound at HS2. This clearly shows that a larger distance between the two domains is preferred when EGCG binds at HS3 of Uba1 as shown by conformations 5 and 6 from Fig. 8B, while a shorter distance is energetically preferred when EGCG binds at HS2 of Uba1.

### Molecular mechanics Poisson–Boltzmann surface area (MM/PBSA) end-point free energy estimates of EGCG binding to Uba1

A simple calculation of binding affinity in terms of the free-energy estimate of EGCG binding with Uba1 at HS2 and at HS3 has been summarized in Table 2.

**Table 2:** Summary of the binding results obtained from the MM/PBSA analysis

	Van der Waals contribution (kcal mol^−1^)	Electrostatic energy contribution (kcal mol^−1^)	Electrostatic contribution to the solvation free energy (kcal mol^−1^)	Δ*G*_total_ (kcal mol^−1^)
EGCG binding at HS2	−40.11	−67.86	91.08	−20.89
EGCG binding at HS3	−32.96	−88.60	112.24	−15.16

The MM/PBSA method has not yet achieved complete accuracy as it involves several severe approximations, but it is still used commonly for pre-screening of viable drug candidates into actives or inactives. This method cannot distinguish between ligands that differ by less than an order of magnitude in binding affinity, which is <6 kJ mol^−1^ or 1.43 kcal mol^−1^. Despite the inaccuracy, our results show a large difference between binding affinities of EGCG at HS2 and HS3 post-simulation. From these values, it is clear that binding of EGCG at HS2 with −20.89 kcal mol^−1^ is higher than EGCG at HS3 with −15.16 kcal mol^−1^.

## Discussion

Upon screening small-molecule libraries in an *in vitro* assay for inhibition of the ubiquitination of proliferating cell nuclear antigen and step-specific assays for individual components of the pathway, we identified the green tea catechin EGCG as an inhibitor of Uba1∼ubiquitin thioester formation with IC_50_ values for Uba1 inhibition of 1.63 μM by a gel-based assay and 0.49 μM by an amplified luminescent proximity homogeneous assay (Alpha).^1,2^ EGCG targets Uba1 directly, reversibly and inhibits the activity of Uba1 both *in vitro* and in cells.^1,2^ We conducted SAR studies with a range of related compounds, among them ECG and EGC; we found that ECG was slightly less potent in activity against Uba1 with IC_50_ values of 4.22 μM by the gel-based assay and 0.77 μM by the Alpha system than EGCG, while EGC was even less active against Uba1 with IC_50_ values of 7.58 μM by the gel-based assay and 5.96 μM by the Alpha system.^1,2^

We were curious about the mode of binding of EGCG, ECG, and EGC and hoped to gain some insight into their mechanisms of action. To address these questions, we used a molecular docking approach with Uba1 and the ligands EGCG, ECG, and EGC at four previously reported hot spots (HS1–HS4) in the Uba1 protein.^28^ We first confirmed that the four positions were likely hot spots in the Uba1 structure with DoGSiteScorer. The docking scores of EGCG, ECG, and EGC to the Uba1 structure suggested that the HS2 and HS3 are the target sites where the three compounds are most likely to bind. The order of docking scores at HS2 fits with our published experimental results while docking scores for HS3 are nearly the same and highest. We can reasonably infer that EGCG, ECG, and EGC may bind HS2 and/or HS3 and thus block Uba1∼ubiquitin thioester formation.

Furthermore, to understand the effects of EGCG binding Uba1 at the two most plausible sites, an aMD simulation protocol was employed. We observed that EGCG binding at HS2 induces a hinge-like domain motion in the Uba1 protein, where the SCCH and UFD domains come into close proximity, resulting in closure of the ubiquitin-binding site. This is a plausible mechanism for inhibition of Uba1∼ubiquitin thioester formation. Such conformational changes of the individual domains of Uba 1 occur with Uba1∼ubiquitin thioester formation which requires the active site cysteine to be in close proximity to the C-terminus of ubiquitin; in the *S. cerevisiae* Uba1 structure,^39^ Cys600 is 35 Å away from the adenylation site, suggesting large conformational adjustments such as hinge motions of the connecting loops that link the AAD, SCCH, and FCCH domains and affect their relative orientations or conformational shift around the cysteine of the SCCH domain, as proposed by Walden *et al.*^40^ Apart from this, the SCCH domain movement is also crucial for disassembly of the AAD domain to transform the Uba1 active site into a supportive state for thioester bond formation. The contacts between the FCCH and SCCH domains, on the one hand, and the IAD and SCCH domains, on the other, are disrupted and new contacts are formed that eventually stabilize this newly attained closed conformation. This rotation of the SCCH domain has been reported for the E1 of the ubiquitin-like modifier SUMO^41,42^ and also a rotation of 106° observed for the *S. pombe* Uba1.^27^ Hann *et al.*^29^ also reported the structures of *S. pombe* Uba1 in two states, where the SCCH domain rotates for 124° from open to closed accompanied by a translation of 0.8 Å.

Moreover, we previously carried out thioester formation experiments by the pre-incubation of either Uba1 or ubiquitin with EGCG.^1^ The pre-incubation of Uba1 alone with EGCG prior to the addition of ubiquitin and ATP leads to inhibition of the Uba1∼ubiquitin thioester adduct, but this is not observed when compound is pre-incubated with ubiquitin first before addition of Uba1, in which case, the inhibition is mitigated. This is consistent with the notion that the rotation of SCCH domain observed during aMD simulations caused by EGCG binding leads to a structural shift in the ubiquitin-binding interfaces.

It is known that residues from the AAD, IAD, and FCCH domains, as well as the crossover loop, are involved in ubiquitin binding as a part of three distinct networks of intermolecular interactions defined as interfaces 1, 2 and 3.^28^ Upon superimposition of the hUba1 structure (PDB code: 6dc6) with the post-simulated Uba1–EGCG complex, we found that no significant structural shift was observed for residues at interface 1 (AAD domain) except a slight rotation of the Phe926 side chain (ESI Fig. 2†). On the other hand, the second interface at the FCCH domain involving Arg239 moves ∼20 Å away from the interacting Asp32–Glu34 patch of ubiquitin while hUba1 Gln243 moves at a similar distance from ubiquitin's Thr12 residue, thereby disrupting interactions at interface 2. Similarly, interface 3, composed of the AAD and the crossover loop, which are possibly involved in guiding ubiquitin's C-terminus to hUba1's active site for catalysis, were also compared through superimposition of the two structures. This revealed that Ser621 and Asp623 of the crossover loop also show a greater distance from ubiquitin's Arg72 when compared to the pre-simulated hUBa1 structure. Another important residue, Gly605 from the AAD domain, possibly involved in creating space for ubiquitin's C-terminus, also shows a slight shift from the original position and replaced by Phe926 side chain of interface 1. This shift would likely crowd the space required for ubiquitin's C-terminus entry at the hUba1 active site.

Moreover, other important stabilizing interactions of the hUba1∼ubiquitin adduct involving Arg74 of ubiquitin with Arg581 of AAD domain and with Glu626 of the crossover loop also shows a shift of ∼15 Å, which likely disrupts hUba1∼ubiquitin adduct formation in the EGCG-bound hUBa1. Originally, the Glu626 side chain is likely involved in a salt-bridge interaction with ubiquitin's Arg74 side chain which otherwise would not be formed at such a large distance. Another important salt bridge between ubiquitin's Arg42 and hUba1 Asp623 of the crossover loop is also disrupted due to a ∼15 Å conformational shift of the crossover loop away from the ubiquitin-binding site.

In our hypothesis, EGCG binding at HS2 of Uba1 causes the SCCH domain rotation before ubiquitin binding and results in the disruption of ubiquitin-stabilizing interfaces involving the FCCH domain and the crossover loop. In experimental support of this, we found the formation of the Uba1∼ubiquitin thioester adduct is inhibited if EGCG is introduced to the Uba1 sample before ubiquitin, but not if EGCG is added to after adding ubiquitin.^1^ Since the gallate esters (EGCG and ECG) are more potent than EGC,^1,2^ which lacks the ester bond, an obvious mechanism suggests itself: *viz.*, that attack of the carbonyl carbon of the ester by a nucleophilic amino acid residue on Uba1 might help explain the higher levels of activity of EGCG and ECG. We found post-simulation poses where Ser1023 is in proximity of the EGCG ester when the distance between UFD and SCCH domain is at the lowest. Since we found that EGCG's inhibition of Uba1 recovers when compound is washed out by serial centrifugation,^1^ any transacylation product would have to be hydrolyzable. It is also possible that the mechanism does not involve covalent modification at all and that the gallate ester is involved in the activity for structural reasons. The only partial loss of inhibitory activity against Uba1 with EGC,^1,2^ however, means that the esterified gallate moiety is not absolutely critical, which suggests the ester would only be partly involved in the mechanisms of inhibition by these catechins.

On the other hand, when EGCG binds to HS3, this domain motion would likely be minor, in contrast to the case of HS2. After aMD, we observed only two amino acid residues (Asp562 and Gln992) that form H-bonds with EGCG pre- or post-simulation suggesting a large conformational change in the structure. Binding to HS3, which shows only minor movement of the SCCH and UFD domains, results in almost the same H-bonding pattern in pre- or post-simulations. While binding at HS2 immediately offers a better explanation for the mechanisms behind the inhibitory effects of the compounds and the experimental SAR results,^1,2^ we cannot rule out alternative or additional concomitant binding to HS3.

## Conclusions

This study highlights that catechins are viable candidates for structure-based rational drug design and can bind with human Uba1 at multiple sites. Based on docking results, HS2 between the UFD and AAD domains and HS3 in the SCCH domain appear to be the most plausible binding sites. An enhanced sampling aMD simulation analysis of EGCG binding at the two sites revealed that binding at HS2 induces a strong conformational change which results in the disruption of the interfaces at the ubiquitin-binding site which, in principle, would inhibit further reactions. EGCG binding at HS3 also results in a small SCCH hinge-like motion but not as strong as seen for the former complex. The binding score at HS2 also follows the experimental rank order of activity of EGCG > ECG > EGC, while at HS3 it is ECG > EGCG > EGC which cannot be overlooked. This study sheds light on possible mechanisms of inhibition of Uba1 by catechin derivatives.

## Author contributions

PG, GF, and CT designed the study and coordinated the initial draft of the manuscript. CT implemented the molecular dynamics simulations and analysis of the complexes. PG, GF, and CT analyzed the results and generated the figures and table. All authors contributed to writing the manuscript. All authors read and approved the final manuscript.

## Conflicts of interest

The authors declare that the research was conducted in the absence of any commercial or financial relationships that could be construed as a potential conflict of interest.

## Supplementary Material

RA-011-D0RA09847G-s001
