# Supplementary material for: Mode of inhibitory binding of epigallocatechin gallate to the ubiquitin-activating enzyme Uba1 via accelerated molecular dynamics
Source: RSC Adv. 2021 Feb 22;11(14):8264–76. doi: 10.1039/d0ra09847g (PMC8695214; doi:10.1039/d0ra09847g)

Electronic Supplementary Material (ESI) for RSC Advances.
This journal is © The Royal Society of Chemistry 2021

Mode of Inhibitory Binding of Epigallocatechin Gallate to the Ubiquitin-Activating Enzyme Uba1 via Accelerated Molecular Dynamics

Paras Gaur[1,2†], Gabriel Fenteany[1,†*], Chetna Tyagi[2,3*]

[1]Institute of Genetics, Biological Research Centre, Szeged, Temesvári krt. 62, 6726 Szeged, Hungary

[2]Doctoral School of Biology, Faculty of Sciences and Informatics, University of Szeged, Közép fasor 52, Szeged, 6726, Hungary

[3]Department of Microbiology, Faculty of Science and Informatics, University of Szeged, Szeged, Közép fasor 52, 6726 Szeged, Hungary

[†]These authors contributed equally to this work.

[*]Correspondence: CT, chetna.tyagi@bio.u-szeged.hu

Supplementary Figure 1. Structural dynamics observed for hUba1 upon EGCG binding (A) A comparative snapshot of the binding pose of EGCG at HS2 before and after aMD simulations. (B) A comparative snapshot of the change in binding pocket upon EGCG binding. (C) The root-mean-square deviation in Å calculated for EGCG when bound at HS2 (in blue) and at HS3 (in red).

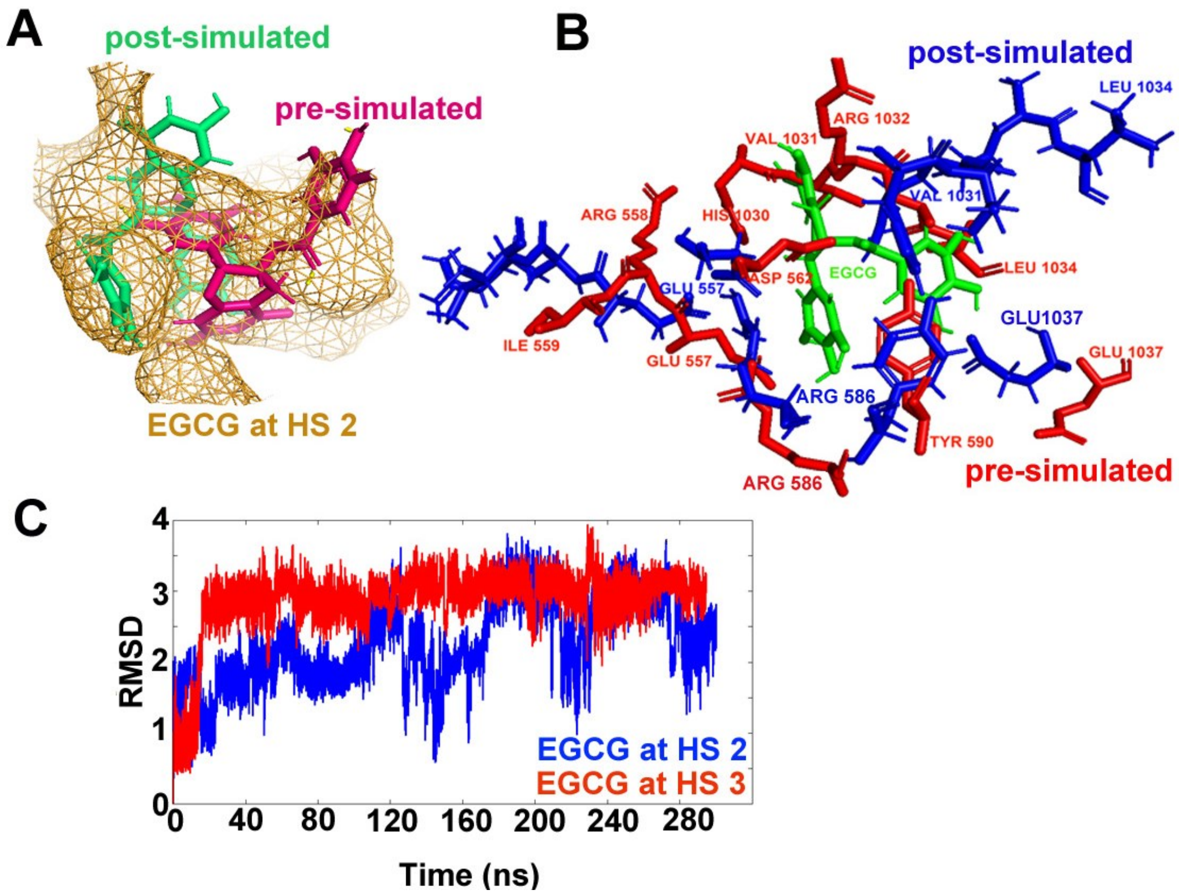

Supplementary Figure 2. Comparison of pre- and post-simulation poses of Uba1 with EGCG bound at HS 2 (A) Uba1~Ub interface 1; small difference observed in the positions of Uba1 Phe926 from Ub Leu8 (in red) (B) Uba1~Ub interface 2; ~20 Å shift in position of Uba1 Arg239 from Ub Asp32-Glu34 patch and (C) Uba1 Gln243 from Ub Thr12 (D) Uba1~Ub interface 3; similar shift in position observed for Uba1 Ser621 and Asp623 of the crossover loop from Ub C-terminal Arg72 (E) crossover loop; shift in position observed for Uba1 Glu626 from Ub C-terminal Arg74 leading to disruption of salt-bridge formation (F) crossover loop; ~15 Å shift in position observed for Uba1 Asp623 from Ub Arg42 leading to disruption of salt-bridge formation.

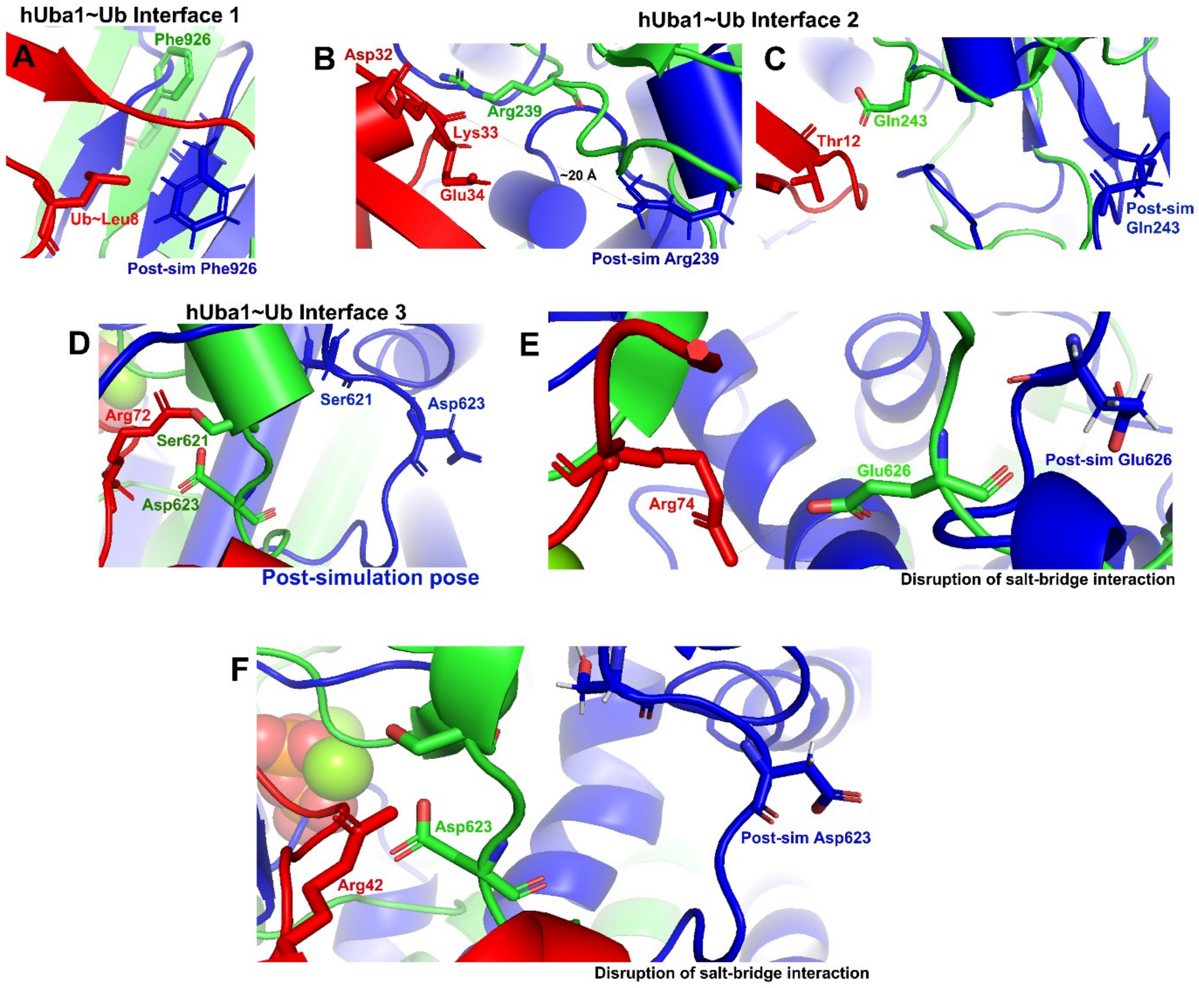

Supplement: RA-011-D0RA09847G-s001 [file RA-011-D0RA09847G-s001.pdf]
